
JOURNAL INFORMATION
==============================
NLM Title Abbreviation: Sci China Life Sci
Journal ID: Sci China Life Sci
NLM Journal ID: 101529880

Science China. Life Sciences

ISSN: 1674-7305
EISSN: 1869-1889

ARTICLE INFORMATION
==============================
PMCID: PMC12678458
PMID: 40705242
DOI: 10.1007/s11427-025-2997-0
Article ID: 2997
Article version: 1
Subjects: Publisher Correction

Publisher Correction to: Identification of a von Willebrand factor type A protein affecting both grain and flag leaf morphologies in wheat

Zhou Chunyun 1
Xiong Hongchun xionghongchun@caas.cn
1
Jia Yong 2
Guo Huijun 1
Fu Meiyu 1
Xie Yongdun 1
Zhao Linshu 1
Gu Jiayu 1
Li Huiyuan 1
Li Yuting 1
Xin Peiyong 3
Chu Jinfang 3 4
Li Chengdao 2
Liu Luxiang liuluxiang@caas.cn
1
1 https://ror.org/0313jb750 grid.410727.7 0000 0001 0526 1937 State Key Laboratory of Crop Gene Resources and Breeding/National Engineering Laboratory of Crop Molecular Breeding/CAEA Research and Development Centre on Nuclear Technology Applications for Irradiation Mutation Breeding, Institute of Crop Sciences, Chinese Academy of Agricultural Sciences, Beijing, 100081 China
2 https://ror.org/00r4sry34 grid.1025.6 0000 0004 0436 6763 Western Crop Genetics Alliance, College of Science, Health, Engineering and Education, Murdoch University, Perth, WA 6150 Australia
3 https://ror.org/034t30j35 grid.9227.e 0000000119573309 National Centre for Plant Gene Research, Institute of Genetics and Developmental Biology, Chinese Academy of Sciences, Beijing, 100101 China
4 https://ror.org/05qbk4x57 grid.410726.6 0000 0004 1797 8419 University of Chinese Academy of Sciences, Beijing, 100049 China
Electronic publication date: 2025 Jul 7
Print publication date: 2025
Volume: 68
Issue: 12
First page: 3800
Last page: 3800
Copyright: © The Author(s) 2025
Copyright year: 2025
License: Open Access This article is licensed under a Creative Commons Attribution 4.0 International License, which permits use, sharing, adaptation, distribution and reproduction in any medium or format, as long as you give appropriate credit to the original author(s) and the source, provide a link to the Creative Commons licence, and indicate if changes were made. The images or other third party material in this article are included in the article’s Creative Commons licence, unless indicated otherwise in a credit line to the material. If material is not included in the article’s Creative Commons licence and your intended use is not permitted by statutory regulation or exceeds the permitted use, you will need to obtain permission directly from the copyright holder. To view a copy of this licence, visit http://creativecommons.org/licenses/by/4.0/.
License URL: https://creativecommons.org/licenses/by/4.0/

Erratum for: Identification of a von Willebrand factor type A protein affecting both grain and flag leaf morphologies in wheat. 3 6 2024 Sci China Life Sci 10.1007/s11427-024-2629-1 38850399
issue-copyright-statement: © Science China Press 2025

==============================
The online version of the original article can be found at 10.1007/s11427-024-2629-1.

